# Elevated Glucose-Potassium Ratio Predicts Preoperative Rebleeding in Patients With Aneurysmal Subarachnoid Hemorrhage

**DOI:** 10.3389/fneur.2021.795376

**Published:** 2022-01-13

**Authors:** Jiayin Wang, Qiangqiang Feng, Yinbin Zhang, Weizhi Qiu, Hongzhi Gao

**Affiliations:** ^1^Department of Neurosurgery, The Second Affiliated Hospital of Fujian Medical University, Quanzhou, China; ^2^Department of Neurosurgery, The Second Affiliated Clinical Medical College of Fujian Medical University, Quanzhou, China; ^3^Department of Neurosurgery, Neurosurgery Research Institute, The First Affiliated Hospital of Fujian Medical University, Fuzhou, China

**Keywords:** glucose, potassium, aneurysm subarachnoid hemorrhage, rebleed, risk factor

## Abstract

**Introduction:** Recent reports revealed that higher serum glucose-potassium ratio (GPR) levels at admission were significantly associated with poor outcomes at 3 months following aneurysmal subarachnoid hemorrhage (aSAH). This study aimed to investigate the association between GPR and the risk of rebleeding following aSAH.

**Methods:** This single-center retrospective study of patients with aSAH was conducted in our hospital between January 2008 and December 2020. Patients meeting the inclusion criteria were divided into the rebleed group and the non-rebleed group. Univariate and multivariate analyses were implemented to assess the association between risk factors of rebleeding and outcomes.

**Results:** A total of 1,367 patients experiencing aSAH, 744 patients who met the entry criteria in the study [mean age (54.89 ± 11.30) years; 60.50% female patients], of whom 45 (6.05%) developed rebleeding. The patients in the rebleed group had significantly higher GPR levels than those of patients without rebleeding [2.13 (1.56–3.20) vs. 1.49 (1.23–1.87); *p* < 0.001]. Multivariable analysis revealed that higher mFisher grade and GPR were associated with rebleeding [mFisher grade, odds ratios (OR) 0.361, 95% CI 0.166–0.783, *p* = 0.01; GPR, OR 0.254, 95% CI 0.13–0.495, *p* < 0.001]. The receiver operating characteristics (ROCs) analysis described that the suitable cut-off value for GPR as a predictor for rebleeding in patients with aSAH was determined as 2.09 (the area under the curve [AUC] was 0.729, 95% CI 0.696–0.761, *p* < 0.0001; the sensitivity was 53.33%, and the specificity was 83.98%). Pearson correlation analysis showed a significant positive correlation between GPR and mFisher grade, between GPR and Hunt–Hess grade (mFisher grade *r* = 0.4271, OR 0.1824, 95% CI 0.3665–0.4842, *p* < 0.001; Hunt–Hess grade *r* = 0.4248, OR 0.1836, 95% CI 0.3697–0.4854, *p* < 0.001). The patients in the poor outcome had significantly higher GPR levels than those of patients in the good outcome [1.87 (1.53–2.42) vs. 1.45 (1.20–1.80); *p* < 0.001]. Multivariable analysis demonstrated that GPR was an independent predictor for poor prognosis. The AUC of GPR was 0.709 (95% CI 0.675–0.741; *p* < 0.0001) (sensitivity = 77.70%; specificity = 55.54%) for poor prognosis.

**Conclusion:** Higher preoperative serum GPR level was associated with Hunt–Hess grade, mFisher grade, rebleeding, and unfunctional outcome, and that they predicted preoperative rebleeding and the 90-days outcome of non-diabetic patients with aSAH, who had potentially relevant clinical implications in patients with aSAH.

## Introduction

Subarachnoid hemorrhage (SAH) from a ruptured intracranial aneurysm contributes to only 5% of stroke cases but occurs at a mean age of 55 years and is associated with a high mortality and disability rate (1, 2). The major early complication is aneurysmal rebleeding with an incidence of 4–13.6% during the first 24 h (3, 4). Early rebleeding is associated with poor-neurosurgical outcomes and mortality rates as high as 50–80% (3, 5). Some studies identified risk factors for preoperative rebleeding have included sex, advanced age, systolic blood pressure >160 mmHg, aneurysm size larger than 10 mm, neurological severity (high Fisher grade and high Hunt–Hess grade), and biomarkers (3–15). Although the mechanism of rebleeding is poorly understood and controversial, studies have pointed the inflammatory response after aSAH may be related to rebleeding (15, 16). When the aneurysm ruptures, inflammation occurs simultaneously, and the inflammation subsequently spreads throughout the brain (16). As the disease progresses, the entire brain continues to experience more severe inflammation, which may exacerbate the occurrence of rebleed. Biomarkers related to inflammation that is readily available on admission may help predict the early risk of a complex rebleeding process (15).

Previous studies reported that a higher serum glucose-potassium ratio (GPR) level at admission was significantly associated with poor outcome or mortality at 3 months following aSAH (17, 18). The existing literature left several crucial unanswered questions about whether the GPR was related to the risk of early brain injury. That is, whether elevated GPR is associated with the risk of vasospasm, delayed cerebral ischemia, and rebleeding. Similarly, the exact underlying mechanism is still unrevealed. Coincidentally, Zhang et al. demonstrate (19, 20) that elevated blood glucose level played a pathological role in active bleeding through inflammation pathway, and was associated with hematoma expansion and prevalence of island sign, blend sign in patients with intracerebral hemorrhage (ICH). A meta-analysis (21) reported that hyperglycemia on admission was related to an increased rate of spontaneous intracranial hemorrhage after mechanical thrombectomy in acute ischemic stroke patients. In addition, hypokalemia caused by aSAH may contribute to the occurrence of rebleeding by the increase of vasoconstrictor receptors and the functional impairment of K_ATP_ channels in cerebrovascular myocytes (22, 23).

Based on the aforementioned theory, we hypothesized that higher preoperative serum GPR levels might correlate with Hunt–Hess grade, modified Fisher grade (mFisher grade), and rebleeding. Moreover, GPR levels predicted rebleeding and 90-days outcomes in non-diabetic patients with aSAH.

## Materials and Methods

### Patient Selection

**Figure 1:**
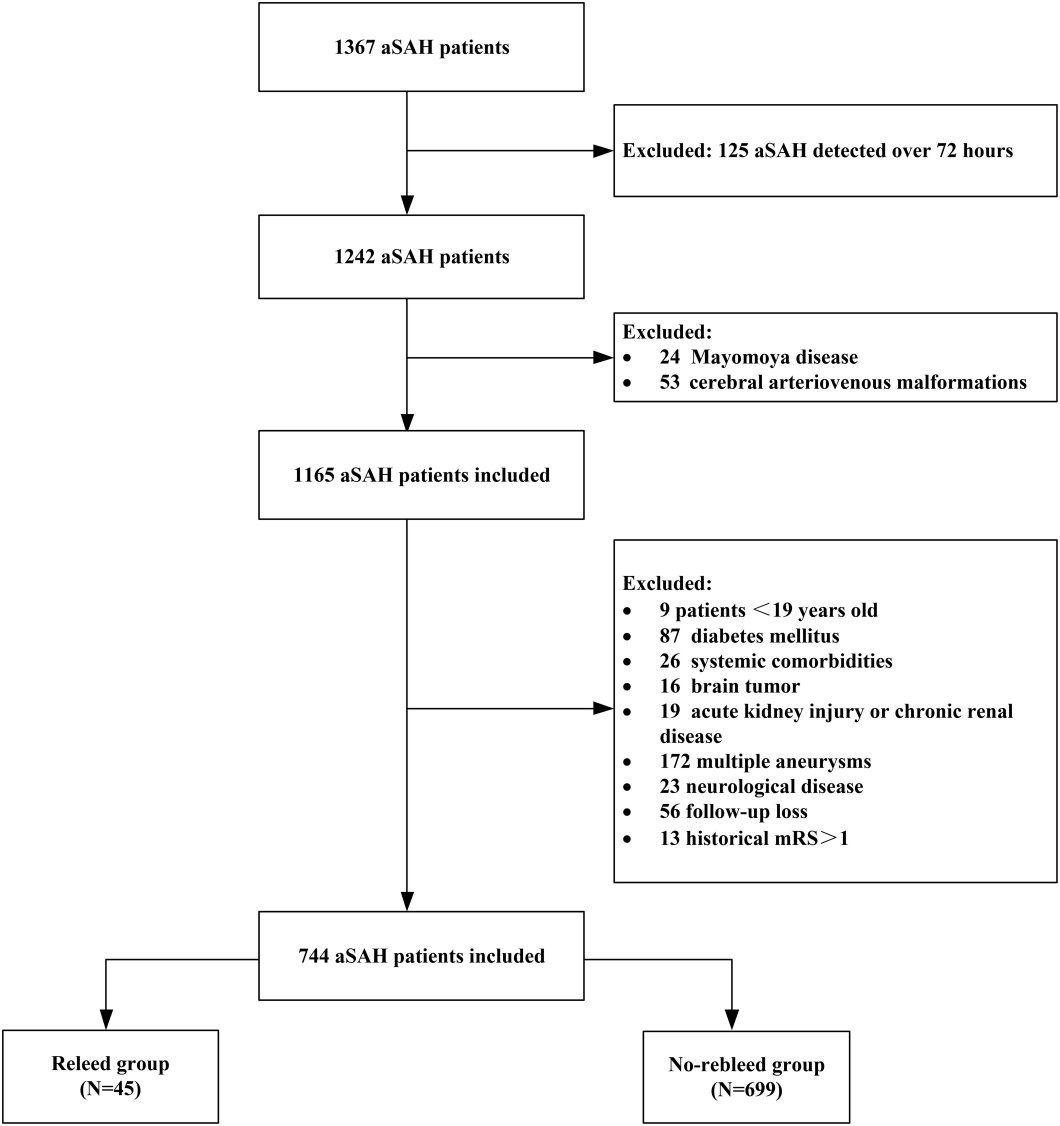
Flow diagram of the patient selection process. aSAH, aneurysmal subarachnoid hemorrhage; mRS, modified Rankin Scale.

This single-center retrospective observational study included all adult patients diagnosed with aSAH who were admitted to the department of neurosurgery in the Second Affiliated Hospital of Fujian Medical University between January 2008 and December 2020. The inclusion criteria included: (1) SAH and rebleeding were diagnosed by CT, and the presence of intracranial aneurysms was confirmed by CT angiography (CTA) or digital subtraction angiography (DSA); (2) baseline CT within 24 h after hemorrhage was performed in all patients, and a follow-up CT scan was performed within 72 h or in cases of the neurological function deterioration; and (3) primary outcome was whether patients developed rebleeding within 72 h following aSAH. The exclusion criteria were as follows: (1) age <19 years; (2) history of diabetes; (3) patients with intracranial tumors, cerebral arteriovenous malformations, moyamoya disease, trauma; (4) multiple intracranial aneurysms; (5) patient had no rebleeding within 72 h after aSAH and received surgical intervention; (6) acute kidney damage or chronic kidney disease; (7) concurrent systemic comorbidities including liver cirrhosis and malignancy; and (8) historical modified Rankin Scale (mRS) > 1. If the patient has rebleeding before surgery, surgical intervention can be performed within 72 h (after rebleeding). This study was approved by the institutional review board of the hospital and carried out in accordance with the principles of the Declaration of Helsinki revised in 2008. The detailed flow of the selection process is given in Figure 1.

### Data Collection

Baseline clinical and demographic characteristics, including age, sex, admission blood pressure, hypertension, time from onset to admission, mFisher scale, Hunt–Hess grade, aneurysm location, admission laboratory, and GPR, were recorded. For the laboratory tests, peripheral venous blood samples were drawn at our neurosurgery department within 1 h of hospitalization. Neurological status was assessed with the Hunt–Hess grade at the initial presentation after bleeding without sedation, and patients were grouped into severe clinical conditions (Hunt–Hess grade 4-5) or mild clinical conditions (Hunt–Hess grade 1-3). Admission CT scans were classified using the modified Fisher grade, and patients were classified as severe aSAH (mFisher grade 3-4) or mild aSAH (mFisher grade 1-2). The clinical prognostic assessment was measured using the mRS score at 90 days (24). The favorable outcome was defined as mRS 0–2, while a poor outcome was mRS 3–6.

### Rebleed

Based on the previous literature (7), we defined rebleeding as a sudden deterioration of neurological function, such as sudden headache and coma, accompanied by radiological signs of increased bleeding compared with previous CT imaging. All the suspected cases of rebleeding underwent the follow-up CT to confirm the clinical diagnosis.

### Statistical Analysis

Continuous variables with normal distribution were presented as mean ± SD and non-normal distribution as median (interquartile range), whereas categorical variables were expressed as count (percentage). The data were presented as a scatter plot with the median. Student's *t*-test or ANOVA carried out comparisons between groups for normally distributed variables, the Mann–Whitney U test and Kruskall-Wallis test for non-normally distributed variables, or chi-squared test for categorical parameters. Analysis of correlation was performed using the Pearson correlation test. Risk factors with *p* < 0.1 in univariable models were entered into the multivariable logistic regression models. GPR was dichotomized as “ ≤ optimal cutoff value” and “> optimal cutoff value” in the multivariate model. The receiver operating characteristics (ROCs) curve and the area under the curve (AUC) were used to assess the sensitivity and specificity of potential parameters for predicting rebleeding. SPSS software (version 25.0, IBM SPSS, IBM Corp, USA), GraphPad Prism 8 (GraphPad Software, San Diego, California, USA), and MedCalc version 20.0.4 (MedCalc Software, Ostend, Belgium, UK) were used for statistical analysis. *p* < 0.05 was considered as statistically significant (ns: *p* > 0.05, ^*^: *p* < 0.05, ^**^: *p* ≤ 0.01, ^***^: *p* ≤ 0.001, ^****^: *p* ≤ 0.0001).

## Results

### Baseline Characteristics of Patients With ASAH

A total of 1,367 patients experiencing aSAH, 744 patients who met the entry criteria in the study [mean age (54.89 ± 11.30) years; 60.50% female patients], of whom 45 (6.05%) developed rebleeding (Table 1 and Figure 1). The time from admission to rebleed was 25 (15.25–35.0) h. A total of 341 patients (45.8%) were in severe clinical condition. A total of 317 patients (42.6%) had severe aSAH on admission. Poor prognosis at 90 days was observed in (18.68%) 139 patients. A total of 744 patients meeting the inclusion criteria were divided into the rebleed group (*n* = 45) and the non-rebleed group (*n* = 699).

**Table 1:** Patient characteristics.

**Variable**	**Value**
Number of patients	744
Rebleed events (*N*, %)	45 (6.05%)
Age (years), mean ±SD	54.89 ± 11.30
**Sex (** ***N*** **, %)**	
Male	294 (39.5)
Female	450 (60.5)
Hypertension (*N*, %)	356 (47.8)
**Hunt-Hess grade (** ***N*** **, %)**	
1–3	403 (54.2)
4–5	341 (45.8)
mFisher grade (*N*, %)	546 (74.8)
1–2	427 (57.4)
3–4	317 (42.6)
**Aneurysm location (** ***N*** **, %)**	
ACA	47 (6.3)
ACoA	238 (32.0)
ICA	123 (16.5)
MCA	149 (20.0)
PCoA	159 (21.4)
Others	28 (3.8)
**Treatment (** ***N*** **, %)**	
Neurosurgical clipping	261 (35.1)
Interventional coiling	483 (64.9)
**Admission laboratory**	
Hb, g/L, mean ± SD	130.10 ± 17.26
HCT, %, mean ± SD	38.67 ± 5.20
PLT, × 10^9^/L, mean ± SD	224.18 ± 67.47
Blood glucose, mmol/L, mean ± SD	5.80 (4.96–7.10)
Serum potassium, mmol/L, mean ± SD	3.93 (3.63–4.19)
GPR, IQR	1.52 (1.23–1.94)
**mRS (** ***N*** **, %)**	
0–2	605 (81.30)
3–6	139 (18.7)

*ACA, anterior cerebral artery; ACoA, anterior communicating artery; aSAH, aneurysmal subarachnoid hemorrhage; GPR, glucose-potassium ratio; Hb, Hemoglobin; HCT, hematocrit; ICA, Internal Carotid Artery; IQR, Interquartile range; MCA, middle cerebral artery; mFisher, modified Fisher; mRS, modified Rankin Scale; OR, Odds ratios; PCoA, posterior communicating artery; PLT, platelet; PT, prothrombin time; SD, Standard deviation; SBP, Systolic blood pressure*.

### Association of GPR With Rebleeding

**Figure 2:**
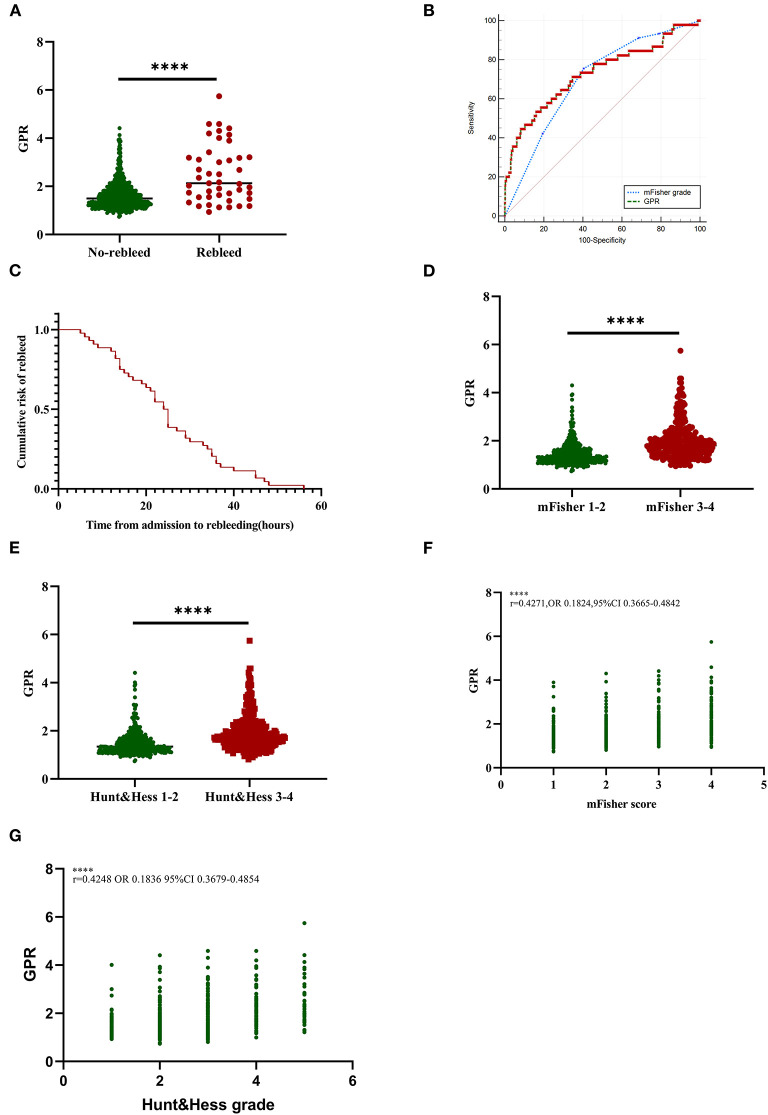
Association of GPR levels with rebleed and initial clinical status in aSAH patients. **(A)** GPR levels in patients with rebleed and non-rebleed. **(B)** The ROC curve analyses comparing admission mFisher grade and GPR for predicting rebleeding in patients with aSAH. The AUC of mFisher grade, GPR were 0.698 (95% CI 0.664–0.731, *p* < 0.0001; the sensitivity was 75.56% and the specificity was 59.51%), 0.729 (95% CI 0.696–0.761, *p* < 0.0001; the sensitivity was 53.33% and the specificity was 83.98%). Z-test illustrated that AUC of mFisher grade was slightly lower than that of GPR, but no statistically significant difference existed (*Z* = 0.843, *p* = 0.3993). **(C)** Cumulative rebleeding rate using the Kaplan–Meier analysis within 72 h after admission in the rebleed group. **(D)** GPR levels in patients with mild (mFisher1-2) and severe (mFisher3-4) aSAH. **(E)** GPR levels in patients with mild (Hunt-Hess grade 1-3) and severe (Hunt-Hess grade 4-5) clinical conditions. **(F)** The correlation of GPR levels with mFisher grade. **(G)** The correlation of GPR levels with Hunt–Hess grade. Median with IQR was shown for scatter-dot plot in **(A,D,E)**. Groups were compared using the Mann–Whitney *U* tests. Correlations were determined using Pearson's correlation analysis. aSAH, aneurysmal subarachnoid hemorrhage; AUC, area under the curve; GPR, glucose-potassium ratio; mFisher, modified Fisher; ROC, receiver operating characteristic. ****p ≤ 0.0001.

Demographic characteristics, clinical features, and laboratory data of patients with aSAH in the two groups were summarized in Table 2. There were significant statistical differences in Hunt–Hess grade, mFisher grade, blood glucose, serum potassium concentration, and GPR in univariate analysis between the rebleed group and the non-rebleed group. Higher blood glucose levels were detected in patients with rebleeding compared with those without [8.22 (5.80–10.52) vs. 5.74 (4.94–6.90) mmol/l, *p* < 0.001; Table 2], while lower serum potassium [3.71 (3.34–4.01) vs. 3.94 (3.65–4.20) mmol/l, *p* < 0.001; Table 2]. The patients in the rebleed group had significantly higher GPR than those of patients without rebleeding [2.13 (1.56–3.20) vs. 1.49 (1.23–1.87); *p* < 0.001] (Figure 2A). Adjustment of OR was made for all outcomes on the SBP, mFisher grade, Hunt–Hess grade, serum calcium, and higher mFisher grade and GPR were associated with rebleeding (mFisher grade OR 0.361, 95% CI 0.166–0.783, *p* = 0.01; GPR, OR 0.254, 95% CI 0.13–0.495, *p* < 0.001; Table 2), while Hunt–Hess grade was not associated with rebleeding.

**Table 2:** Univariate and multivariate analysis of risk factors related to rebleeding in patients with aSAH.

**Characteristics**	**Non-rebleed** **(*N* = 699)**	**Rebleed** **(*N* = 45)**	**Univariate** ***P* value**	**Multivariate analysis**		
				**OR**	**95%CI**	***P*** **value**
Age (yrs), mean ±SD	54.92 ± 11.28	54.38 ± 11.74	0.764			
Sex (*N*, %)			0.485			
Male	274 (39.2)	20 (44.4)				
Female	425 (60.8)	25 (55.6)				
SBP, mmHg, mean ±SD	142.42 ± 25.12	149.90 ± 26.62	0.072	1.001	0.989–1.013	0.876
DBP, mmHg, mean ±SD	84.24 ± 14.11	85.87 ± 11.07	0.353			
Hypertension (N, %)	332 (47.5)	24 (53.3)	0.447			
Hunt-Hess grade			0.04	0.841	0.406–1.744	0.642
1–3	338 (55.5)	15 (33.3)				
4–5	311 (44.5)	30 (66.7)				
mFisher grade			<0.001	0.361	0.166–0.783	0.01
1–2	416 (59.5)	11 (24.4)				
3–4	283 (40.5)	32 (75.6)				
Aneurysm location			0.740			
ACA	43 (6.2)	4 (8.9)				
ACoA	223 (31.9)	15 (33.3)				
ICA	114 (6.3)	9 (20.0)				
MCA	139 (19.9)	10 (22.2)				
PCoA	153 (21.9)	6 (13.3)				
Others	27 (3.9)	1 (2.2)				
Time from onset to admission, h, IQR	15 (10, 17)	12 (6, 17)	0.145			
**Admission laboratory**						
Hb, g/L, mean ±SD	130.09 ± 16.91	130.24 ± 22.25	0.963			
HCT, %, mean ±SD	38.67 ± 5.12	38.74 ± 6.28	0.941			
PLT, × 10^9^/L, mean ±SD	224.11 ± 67.54	225.29 ± 67.13	0.910			
Blood glucose, mmol/L, IQR	5.74 (4.94,6.90)	8.22 (5.80,10.52)	<0.001			
Serum potassium, mmol/L, IQR	3.94 (3.65,4.20)	3.71 (3.34,4.01)	<0.001			
GPR, IQR	1.49 (1.23,1.87)	2.13 (1.56,3.20)	<0.001	0.254	0.13–0.495	<0.001
Serum sodium, mmol/L, IQR	140.80 (138.30,143.0)	140.4 (137.0–143.15)	0.593			
Serum calcium,mmol/L, IQR	2.18 (2.09,2.26)	2.15 (2.05,2.24)	0.095	0.589	0.072–4.802	0.621
**Coagulation function**						
PT,seconds, mean ±SD	12.19 ± 1.43	12.30 ± 1.18	0.562			
aPTT,seconds, mean ±SD	29.79 ± 6.12	30.31 ± 4.31	0.452			
FIB, g/L, mean ±SD	3.12 ± 1.77	3.18 ± 1.10	0.826			
mRS (N, %)			<0.001			
0–2	608 (87.0)	15 (33.3)				
3–6	91 (13.0)	30 (66.7)				

*ACA, anterior cerebral artery; ACoA, anterior communicating artery; aPTT, Activated partial thromboplastin time; aSAH, aneurysmal subarachnoid hemorrhage; CI, confidence interval; DBP, Diastolic blood pressure; FIB, Fibrinogen; GPR; glucose-potassium ratio; Hb, Hemoglobin; HCT, hematocrit; ICA, Internal Carotid Artery; IQR, Interquartile range; MCA, middle cerebral artery; mFisher, modified Fisher; mRS, modified Rankin Scale; OR, Odds ratios; PCoA, posterior communicating artery; PLT, platelet; PT, prothrombin time; SD, Standard deviation; SBP, Systolic blood pressure*.

The ROC analysis described that the suitable cutoff value for GPR as a predictor for rebleeding in patients with aSAH was determined as 2.09 (the AUC was 0.729, 95% CI 0.696–0.761, *p* < 0.0001; the sensitivity was 53.33%, and the specificity was 83.98%; Figure 2B). The predictive performances of the mFisher grade (the AUC was 0.698, 95% CI 0.664–0.731, *p* < 0.0001; the sensitivity was 75.56%, and the specificity was 59.51% based on the best threshold of two) was also illustrated by the ROC analysis. The AUC of GPR levels and the AUC of mFisher grade were comparable by *Z*-test and the AUC of mFisher grade was slightly lower than that of GPR, but no statistically significant difference existed (*Z* = 0.843, *p* = 0.3993; Figure 2B). Cumulative rebleeding rate using Kaplan-Meier analysis within 72 h after admission in the rebleed group is present in Figure 2C.

### Association of GPR With Initial Clinical Status at Admission

In order to investigate the relationship between different levels of GPR and rebleeding, we grouped the patients based on the quartile range of GPR levels. Of all the clinical and radiological variables, age, Hunt–Hess grade, mFisher grade, rebleed, and poor outcomes were significantly different among different levels of GPR (*p* < 0.05; Table 3). GPR ≥ 1.941 was found in 183 patients (24.60%) and observed in 24 patients with rebleeding(14.2%) (Table 3). GPR was significantly higher in patients with aSAH with mFisher 3–4 compared with mFisher 1–2 [1.81 (1.49–2.28) vs. 1.33 (1.16–1.62), *p* < 0.0001; Figure 2D]. In addition, patients with the severe clinical condition on admission (Hunt–Hess 4-5) had a higher GPR [1.76 (1.44–2.17) vs. 1.34 (1.16–1.65), Figure 2E]. Pearson correlation analysis showed a significant positive correlation between GPR and mFisher grade, between GPR and Hunt–Hess grade (mFisher grade *r* = 0.4271, OR 0.1824, 95% CI 0.3665–0.4842, *p* < 0.001; Hunt–Hess grade *r* = 0.4248, OR 0.1836, 95% CI 0.3697–0.4854, *p* < 0.001) (Figures 2F,G).

**Table 3:** Patient's demographics and baseline characteristics by GPR.

**Characteristics**	**GPR** **(≤1.235) (*N* = 192)**	**GPR** **(1.235–1.522) (*N* = 183)**	**GPR** **(1.522–1.941) (*N* = 186)**	**GPR** **≥1.941 (*N* = 183)**	***P*** **value**
Age (yrs), mean ± SD	52.90 ± 12.07	55.30 ± 10.88	54.92 ± 11.32	56.49 ± 10.61	0.019
**Sex (** ***N*** **, %)**					
Male	88 (45.8)	73 (39.9)	78 (41.9)	55 (30.1)	0.015
Female	104 (54.2)	110 (60.1)	108 (58.1)	128 (69.9)	
SBP, mmHg, mean ±SD	134.08 ± 20.42	138.30 ± 22.04	145.80 ± 25.74	153.70 ± 27.91	<0.001
DBP, mmHg, mean ±SD	82.28 ± 12.94	82.83 ± 12.61	84.93 ± 14.49	87.41 ± 15.14	0.001
Hypertension (N, %)	60 (31.2)	85 (46.4)	93 (50.0)	118 (64.5)	<0.001
**Hunt-Hess grade**					<0.001
1–3	146 (76.0)	121 (66.1)	83 (44.6)	53 (29.0)	
4–5	46 (24.0)	62 (33.9)	103 (55.4)	130 (71.0)	
**mFisher grade (** ***N*** **, %)**					
1–2	158 (82.3)	128 (69.9)	95 (51.1)	46 (25.1)	<0.001
3–4	34 (17.7)	55 (30.1)	91 (48.9)	137 (74.9)	
**Aneurysm location**					0.042
ACA	11 (5.7)	11 (6.0)	11 (5.9)	14 (7.7)	
ACoA	54 (28.1)	51(27.9)	77 (41.4)	56 (30.6)	
ICA	45 (23.4)	31 (16.9)	20 (10.8)	27 (14.8)	
MCA	36 (18.8)	37 (20.2)	36 (19.4)	40 (21.9)	
PCoA	41 (21.4)	40 (21.9)	38 (20.4)	40 (21.9)	
Others	5 (2.6)	13 (7.1)	4 (2.2)	6 (3.3)	
Time from onset to admission, h, IQR	15(11, 17)	14 (10, 17)	14 (9, 18)	15 (8, 18)	0.249
Rebleed (*N*, %)	7 (3.6)	3 (1.6)	9 (4.8)	26 (14.2)	<0.001
**Admission laboratory**					
Hb, g/L, mean ± SD	132.57 ± 16.84	130.38 ± 16.62	129.01 ± 17.0	128.33 ± 18.39	0.084
HCT, %, mean ± SD	39.48 ± 4.63	38.91 ± 5.98	38.23 ± 4.79	38.04 ± 5.24	0.028
PLT, × 10^9^/L, mean ± SD	222.51 ± 69.22	228.72 ± 67.13	215.96 ± 65.73	218.22 ± 66.64	0.033
Blood glucose, mmol/L, IQR	4.70 (4.48, 4.94)	5.38 (5.05, 5.74)	6.41 (5.90, 6.88)	8.62 (7.77, 10.27)	<0.001
Serum potassium, mmol/L, IQR	4.21 (3.99, 4.46)	3.98 (3.70, 4.21)	3.77 (3.59, 4.03)	3.67 (3.35, 3.96)	<0.001
Serum sodium, mmol/L, IQR	141 (139.13, 143.0)	141(139.0, 143.0)	140.15 (137.68, 142.43)	140.0 (137.70–143.0)	0.01
Serum calcium, mmol/L, IQR	2.21 (2.14, 2.30)	2.20 (2.11, 2.27)	2.15 (2.06, 2.22)	2.14 (2.06, 2.24)	<0.001
**Coagulation function**					
PT, seconds, mean ± SD	12.37 ± 2.08	12.22 ± 1.14	12.16 ± 1.01	12.04 ± 1.11	0.150
aPTT, seconds, mean ± SD	31.61 ± 5.90	30.72 ± 5.52	28.90 ± 6.51	28.0 ± 5.48	<0.001
FIB, g/L, mean ± SD	3.20 ± 1.72	3.33 ± 2.34	3.02 ± 1.52	2.93 ± 1.13	0.125
mRS (*N*, %)					<0.001
0–2	178 (92.7)	163 (89.1)	147 (79.0)	117 (63.9)	
3–6	14 (7.3)	20 (10.9)	39 (21.0)	66 (36.1)	

*ACA, anterior cerebral artery; ACoA, anterior communicating artery; aPTT, Activated partial thromboplastin time; aSAH, aneurysmal subarachnoid hemorrhage; CI, confidence interval; DBP, Diastolic blood pressure; FIB, Fibrinogen; GPR; glucose-potassium ratio; Hb, Hemoglobin; HCT, hematocrit; ICA, Internal Carotid Artery; IQR, Interquartile range; MCA, middle cerebral artery; mFisher, modified Fisher; mRS, modified Rankin Scale; OR, Odds ratios; PCoA, posterior communicating artery; PLT, platelet; PT, prothrombin time; SD, Standard deviation; SBP, Systolic blood pressure*.

### Relationship Between Elevated GPR and Poor Outcome

**Figure 3:**
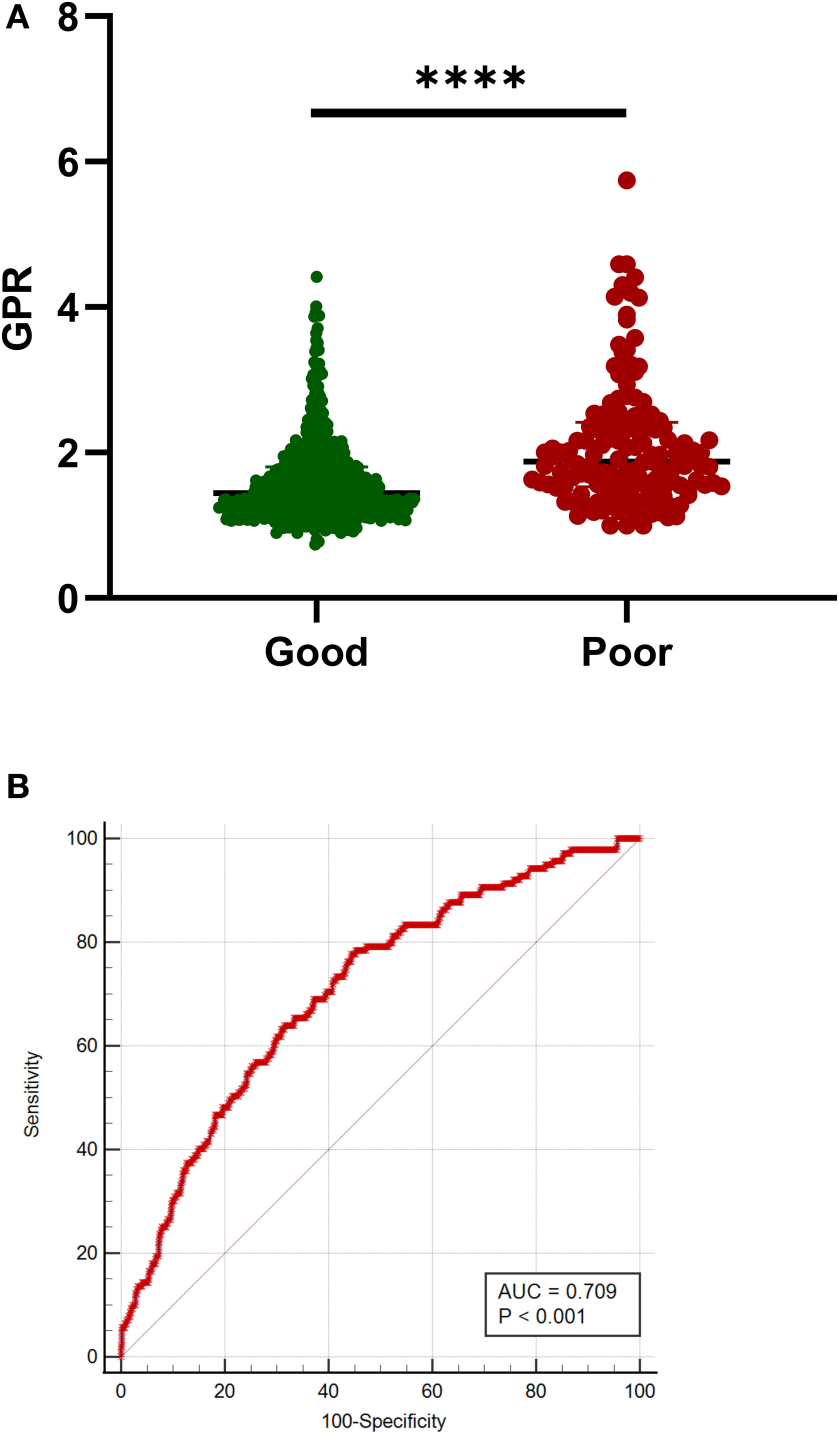
Association of GPR with outcome in patients with aSAH. **(A)** G. GPR levels in patients with good (mRS 0-2) and poor (mRS 3-6) outcomes. **(B)** The ROC curve analysis of GPR for predicting poor prognosis. The AUC of GPR was 0.709 (sensitivity = 77.70%; specificity = 55.54%) for poor prognosis based on a cutoff value of 1.51. Median with IQR was shown for scatter-dot plot in **(A)**. Groups were compared using Mann–Whitney *U* tests. aSAH, aneurysmal subarachnoid hemorrhage; AUC, area under the curve; GPR, glucose-potassium ratio; mRS, modified Rankin Scale; ROC, receiver operating characteristic. ****p ≤ 0.0001.

A total of 605 patients with aSAH (81.32%) had a good prognosis, whereas 139 patients (18.68%) had a poor prognosis. Significant statistical differences were detected in the age, SBP, diastolic blood pressure (DBP), hypertension, Hunt–Hess grade, mFisher grade, blood glucose, serum potassium concentration, serum calcium, aPTT, GPR, and rebleed in univariate analysis. The patients in the poor outcome had significantly higher GPR levels than those of patients in the good outcome [1.87 (1.53–2.42) vs. 1.45 (1.20–1.80); *p* < 0.001; Table 4 and Figure 3A]. Adjustment of OR was made for all outcomes on the age, SBP, DBP, hypertension, Hunt–Hess grade, mFisher grade, serum calcium, aPTT, rebleed (Table 4). The multivariable analysis demonstrated that higher mFisher grade (3-4), higher Hunt–Hess (4-5), rebleed, and GPR were independent predictors for poor prognosis (Table 4). The AUC of GPR was 0.709 (95% CI 0.675–0.741; *p* < 0.0001) (Sensitivity = 77.70%; Specificity = 55.54%) for poor outcome based on a cutoff value of 1.51 (Figure 3B).

**Table 4:** Univariate and multivariate analysis of risk factors related to outcome in patients with aSAH.

**Characteristics**	**Good outcome** **(*N* = 605)**	**Poor outcome** **(*N* = 138)**	**Univariate** ***P*** **value**	**Multivariate analysis**		
				**OR**	**95%CI**	***P*** **value**
Age (yrs), mean ± SD	54.30 ± 11.03	57.46 ± 12.11	0.003	1.022	1.001–1.043	0.037
Sex (*N*, %)			0.254			
Male	245 (40.5)	49 (35.3)				
Female	360 (59.5)	90 (64.7)				
SBP, mmHg, mean ±SD	140.63 ± 24.25	152.61 ± 27.29	0.005	1.004	0.993–1.016	0.480
DBP, mmHg, mean ±SD	83.62 ± 13.96	87.47 ± 13.51	<0.001	1.002	0.983–1.022	0.822
Hypertension (*N*, %)	269 (44.5)	87 (62.6)	<0.001	0.945	0.563–1.587	0.831
Hunt-Hess grade			<0.001	0.443	0.263–.0744	0.002
1–3	369 (61.0)	34 (24.5)				
4–5	236 (39.0)	105 (75.5)				
mFisher grade			<0.001	0.262	0.159–0.431	<0.001
1–2	398 (65.8)	29 (20.9)				
3–4	207 (34.2)	110 (79.1)				
Aneurysm location			0.305			
ACA	40 (6.6)	7 (5.1)				
ACoA	183 (30.2)	55(39.9)				
ICA	99 (16.3)	24 (17.4)				
MCA	124 (20.5)	25 (18.0)				
PCoA	134 (22.1)	25 (18.0)				
Others	25 (4.1)	3 (2.2)				
Time from onset to admission, h, IQR	15 (10, 17)	14 (9, 17)	0.471			
**Admission laboratory**						
Hb, g/L, mean ± SD	129.72 ± 16.92	131.73 ± 18.65	0.245			
HCT, %, mean ± SD	38.50 ± 4.79	39.40 ± 6.65	0.133			
PLT, × 10^9^/L, mean ± SD	223.35 ± 67.30	227.80 ± 68.33	0.489			
Blood glucose, mmol/L, IQR	5.61 (4.86, 6.73)	6.90 (5.97, 8.88)	<0.001			
Serum potassium, mmol/L, IQR	3.96 (3.65, 4.21)	3.79 (3.47, 4.07)	<0.001			
GPR, IQR	1.45 (1.20, 1.80)	1.87 (1.53, 2.42)	<0.001	0.572	0.347–0.944	0.029
Serum sodium, mmol/L, IQR	140.80 (138.30, 142.8)	140.7 (138.0–143.9)	0.277			
Serum calcium,mmol/L, IQR	2.18 (2.10, 2.27)	2.14 (2.05, 2.22)	0.001	0.287	0.066–1.257	0.098
**Coagulation function**						
PT,seconds, mean ± SD	12.21 ± 1.47	12.14 ± 1.16	0.505			
aPTT,seconds mean ± SD	30.05 ± 6.21	28.84 ± 5.09	0.016	0.999	0.963–1.035	0.936
FIB, g/L, mean ± SD	3.11 ± 1.66	3.19 ± 2.03	0.640			
Rebleed (*N*, %)	15 (2.5)	30 (21.6)	<0.001	0.122	0.058–0.258	<0.001

*ACA, anterior cerebral artery; ACoA, anterior communicating artery; aPTT, Activated partial thromboplastin time; aSAH, aneurysmal subarachnoid hemorrhage; CI, confidence interval; DBP, Diastolic blood pressure; FIB, Fibrinogen; GPR, glucose-potassium ratio; Hb, Hemoglobin; HCT, hematocrit; ICA, Internal Carotid Artery; IQR, Interquartile range; MCA, middle cerebral artery; mFisher, modified Fisher; mRS, modified Rankin Scale; OR, Odds ratios; PCoA, posterior communicating artery; PLT, platelet; PT, prothrombin time; SD, Standard deviation; SBP, Systolic blood pressure*.

## Discussion

We presented a single-center retrospective observational study of non-diabetic patients with aSAH that identified elevated admission GPR level as an independent risk factor for rebleeding. Furthermore, GPR also exhibited a comparable performance to mFisher grade in predicting rebleeding. In addition, we have also demonstrated significant correlations between GPR and the amount of intracranial blood produced by initial bleeding, measured by the mFisher grade, and the severity of clinical symptoms, measured by the Hunt–Hess grade. The correlation between elevated GPR levels and increased severity and outcome in patients with aSAH was observed. This is the first study to investigate the potential predictive power of elevated admission GPR levels for rebleeding in patients with aSAH to the best of our knowledge.

Previous literature reported that higher serum GPR level at admission was significantly associated with poor outcome or mortality at 3 months following aSAH (17, 18). However, the studies fail to answer whether the GPR levels are related to early brain injury risk. Similarly, the exact underlying mechanism is still unrevealed. Interestingly, a 3.94-fold difference in the risk of rebleeding and a 4.95-fold difference in unfavorable outcomes between the lowest GPR (≤1.235) and the highest GPR (≥1.941) quartiles (Table 3) was detected in this study. Therefore, this study identifies that elevated admission GPR level is an independent risk factor for rebleeding following aSAH and rebleeding is associated with poor functional outcomes.

Preoperative aneurysm rebleeding has traditionally been a significant reason contributing to morbidity and mortality after aSAH. Previously identified risk factors for preoperative rebleeding have included sex, systolic blood pressure > 160 mm Hg, high Fisher grade, aneurysm characteristics (location, size, and numbers), hyperglycemia, neurological severity (5–9, 11–13, 25–29). Rebleeding following aSAH is a complex and multifactorial event. The pathophysiological mechanisms of rebleeding are still not fully understood and may involve complex interactions between blood products, vasoactive substances, and inflammatory cascades. Previous literature suggests that a higher neutrophile lymphocyte ratio predicts the occurrence of rebleeding, indicating that direct and reactive inflammation may contribute to rebleeding following aSAH. Zhang et al. demonstrate that elevated blood glucose level plays a pathological role in active bleeding through inflammation pathway, and is associated with hematoma expansion and prevalence of island sign, blend sign in patients with ICH (19, 20). Coincidentally, this study also detects that the rebleed group has a higher blood glucose level than that of the non-rebleed group in patients with aSAH. In addition, hypokalemia caused by aSAH may contribute to the occurrence of rebleeding by the increase of vasoconstrictor receptors and the functional impairment of K_ATP_ channels in cerebrovascular myocytes (22, 23). Taken together, we propose that elevated GPR level at admission can predict early rebleeding following aSAH, and it is verified in our study.

Associations of elevated GPR levels with poor outcome and mortality have been described in patients with aSAH (17, 18) but never concerning rebleeding occurrence. How can elevated GPR levels (increased blood glucose and decreased potassium) cause rebleeding? Hyperglycemia often accompanies aneurysm rupture within 72 h after onset. Under stress and injuries, this hyperglycemia directly results from raised blood catecholamine levels or increasing glucagon secretion and inhibiting insulin secretion indirectly (27). Disturbance of glucose metabolism after aSAH reflects the degree of early brain damage, and the brain inflammatory response begins when the aneurysm ruptures (16). Venditti et al. reported that increased glucose levels could lead to a downstream microvascular thrombo-inflammation, blood-brain barrier disruption, and hemorrhagic transformation after thrombectomy in stroke (30). The detrimental effects of hyperglycemia accelerate the destruction of the blood–brain barrier, impair the integrity of the surrounding blood vessels, and promote continuous or emerging bleeding. Inflammation caused by hyperglycemia and the production of oxygen free radicals are also involved in the progress of aSAH and it is reported to be involved in the pathophysiological process of microvascular integrity damage and early rebleeding or hematoma expansion.

Furthermore, it is well-known that the inflammatory process of the aneurysm wall promotes the growth and rupture of intracerebral aneurysms (31–33). The presence and subsequent lysis of red blood cells after aSAH produce an immediate cerebral inflammatory response similar to the inflammatory cascade that characterizes the systemic inflammatory response syndrome (16), leading to local vasoconstriction and hypoxia, which may induce rebleeding. The presence of hyperglycemia and hypokalemia exacerbate the damage as mentioned earlier process.

Potassium is the major intracellular cation, and 98% of the potassium in the human body exists in the intracellular fluid. Potassium is actively transported from plasma to cells by the sodium-potassium adenosine triphosphatase pump (Na/K-ATPase). After aSAH, catecholamines, and insulin reduce serum potassium levels and activate the K_ATP_ channel, inflammation exacerbates this damaging process after aSAH. Reduced potassium may contribute to rebleed by the increase of vasoconstrictor receptors and the functional impairment of K_ATP_ channels in cerebrovascular myocytes (22). Studies from animal models of SAH demonstrated that the K channel is related to the dilation and contraction of the intracranial artery (22, 34). Another recent animal model also indicates that the inflammatory response after SAH leads to oxidative damage to cerebral arteries, weakening ATP-sensitive K channel currents in vascular smooth muscle, and changes in the morphology of basilar arteries (35). Based on this theory, we speculate that lower serum potassium caused by aSAH exacerbates the damage of intracranial arteries. Subsequently, rebleed events occurred on the damaged vessel wall.

The reported literature shows that strict early management of blood glucose, electrolytes, and fluid intake after ICH, can reduce the volume of hematoma growth or the incidence of rebleeding, thereby improving the prognosis (23, 36). Combined with our results, we believe that patients with aSAH with elevated GPR levels can partially reveal a higher risk of active bleeding or rebleeding. In addition, in line with existing literature, a higher mFisher grade is an independent factor in predicting rebleeding after aSAH. It is possible that the amount of bleeding is one of the crucial signs that affect the stability of the aneurysm wall in patients with aSAH after the aneurysm wall ruptures (7, 37). This study indicated that GPR levels also exhibited a comparable performance to mFisher grade in predicting rebleeding following aSAH. Significant correlations between GPR levels, Hunt–Hess grade, and mFisher grade were observed in our study, suggesting that GPR levels can reflect the severity of aSAH to the same extent.

Fujiki et al. (18). reported that a high serum GPR in patients with aSAH would help predict a poor prognosis. Jung et al. (17)also suggested that higher plasma GPR levels on admission were considered a potential predictor of 3-month mortality in patients with aSAH. Likewise, our study demonstrated high-serum GPR level at admission in patients who developed rebleeding and was associated with unfavorable outcomes in patients with aSAH.

However, this study has several limitations, including those inherent to most retrospective observational studies, such as less power to estimate the cause-effect and not control all the confounding factors. Furthermore, laboratory data such as blood glucose and serum potassium are the initial values of admission. Dynamic monitoring of laboratory results may improve the accuracy of our research. Further study is needed to determine whether changes in GPR levels over time are related to the onset of rebleeding. Third, serum glucose and potassium levels may be affected by various factors, such as last food intake time, potassium-consuming diuretics, and cortisol, but we did not consider these factors. The management of blood glucose was not described in detail in this study. Fourth, to observe early rebleeding following aSAH, we excluded patients with aSAH undergoing emergency surgery within 72 h, resulting in a reduction in sample size, which might affect the accuracy of our study. Last but not least, other relevant inflammation markers, such as C-reactive protein, neutrophil-to-lymphocyte ratio, and white blood cell count, are related to rebleeding of aSAH. Unfortunately, this study failed to provide a comparison.

## Conclusion

Higher preoperative serum GPR level was associated with Hunt–Hess grade, mFisher grade, rebleeding, and unfunctional outcome, ant that predicted preoperative rebleeding and the 90-days outcome of non-diabetic patients with aSAH, who had potentially relevant clinical implications in patients with aSAH.

## Data Availability Statement

The original contributions presented in the study are included in the article/supplementary material, further inquiries can be directed to the corresponding authors.

## Ethics Statement

The studies involving human participants were reviewed and approved by the Ethics Committee of the Second Affiliated Hospital of Fujian Medical University. The patients/participants provided their written informed consent to participate in this study.

## Author Contributions

JW and YZ: designed the study, drafted the manuscript, prepared the figures, and interpreted the results. QF and WQ: collected and analyzed the data. YZ: helped in the statistical analysis and result interpretation. HG: supervised the study and revised manuscript. YZ and HG: were identified as the guarantor of the article and taking responsibility for the integrity of the work as a whole. All the authors read and approved the final version of the manuscript.

## Funding

This study was supported by the Science and Technology Planning Project of Health and Family Planning Commission of Fujian Province (No. 2018-CX-34 to HG) and the Young and Middle-aged Project of Fujian Provincial Department of Education (No. JAT190224 to JW).

## Conflict of Interest

The authors declare that the research was conducted in the absence of any commercial or financial relationships that could be construed as a potential conflict of interest.

## Publisher's Note

All claims expressed in this article are solely those of the authors and do not necessarily represent those of their affiliated organizations, or those of the publisher, the editors and the reviewers. Any product that may be evaluated in this article, or claim that may be made by its manufacturer, is not guaranteed or endorsed by the publisher.
